# Aquafeed Enrichment with *Dictyota Dichotoma* and *Bacillus Coagulans*: A Synergistic Strategy to Promote Growth, Immune Function, and Antioxidant Defensive Pathways in *Cyprinus Carpio*

**DOI:** 10.1007/s10126-025-10567-1

**Published:** 2026-02-04

**Authors:** Karim E. A. Khalil, Mohamed F. Abdelghany, Mohamed M. El-Nawsany, Xiaolin Meng, Mohammed A. E. Naiel

**Affiliations:** 1 Fish Production Department, Faculty of Agriculture, Al-Azhar University, P.O.Box 11651, Cairo, Egypt; 2 College of Fisheries, Henan Normal University, Xinxiang, 453007 PR China; 3 Engineering Technology Research Center of Henan Province for Aquatic Animal Cultivation, Henan Normal University, Xinxiang, 453007 PR China; 4 Animal Production Department, Faculty of Agriculture, Zagazig University, Zagazig, 44519 Egypt

**Keywords:** Brown macroalgae, Probiotic, Functional additives, Growth, Immune responses, Oxidative modulation, *Cyprinus carpio*

## Abstract

This study evaluated the effects of dietary supplementation with *Bacillus coagulans* and/or the brown macroalga *Dictyota dichotoma* on growth, blood biochemical indices, immune responses, redox status, and gene regulation in common carp (*Cyprinus carpio*). A total of 400 fingerlings (initially weighing, 3.0 ± 0.03 g) were randomly divided into 40 hapas (10 fish per hapa). These hapas were assigned into equal 8 experimental groups (each group contain five replicates). The groups included: a control (no supplementation), *B. coagulans* alone (2 g/kg), *D. dichotoma* at 2.5, 5, and 10 g/kg (D_1_, D_2_, D_3_), and combinations of *B. coagulans* (2 g/kg) with each level of *D. dichotoma* (BC*D_1_, BC*D_2_, BC*D_3_). After 8 weeks of feeding, the results indicated that the diet supplemented with 10 g/kg of *D. dichotoma* (D3), either alone or in combination with BC, significantly enhanced growth performance, feed conversion ratio (FCR), and feed efficiency ratio (FER). Meanwhile, the fish group fed 2 g/kg of BC showed a notable improvement in feed utilization parameters, although its impact on growth was limited. The whole-body chemical analysis results showed that either BC or *D. dichotoma* alone significantly increased ash content while decreasing protein and lipid levels in a dose-dependent manner. In contrast, the combined BC*D_3_ group significantly enhanced protein retention levels while reducing ash and lipid content. Notably, D3-fed fish showed the highest serum protein content and reduced liver enzyme activities. In contrast, BC alone significantly lowered both liver and kidney health indicators. Additionally, significant interactions between BC and D_3_ were observed for protein fraction levels, AST, and kidney function enzymes, with the combined BC and D3 treatment resulting in the most favorable profile. Furthermore, immunity measurements, including lysozyme, complement C3, IgM, and IgD, improved significantly with the combined supplementation of BC and a high level of *D. dichotoma*. Additionally, the dietary administration of both BC and 10 g/kg *D. dichotoma* significantly boosted antioxidant enzyme activities (glutathione peroxidase, catalase, and superoxide dismutase) while reducing MDA levels. Additionally, co-supplementation significantly upregulated hepatic expression of *IL-6*,* IL-10*,* and TNF-2α.* These findings highlight the synergistic potential of dietary supplementation with 2 g/kg *Bacillus coagulans* and 10 g/kg *Dictyota dichotoma* as effective functional feed additives for enhancing growth performance, immune responses, and oxidative stress resistance in common carp aquaculture.

## Introduction

Aquaculture plays a vital role in global food security, with freshwater species such as common carp (*Cyprinus carpio*) representing one of the most cultivated finfish due to their omnivorous diet, rapid growth, and economic importance (Khafaga et al. 2020). However, the intensification of aquaculture systems often exposes fish to chronic environmental stressors, particularly poor water quality, which can compromise immunity, induce oxidative damage, and reduce growth performance (El-Adl et al. 2024). Consequently, there is a huge growing interest towards employing functional feed additives as sustainable and non-antibiotic tools to support fish health, improve physiological resilience, and enhance production efficiency under intensive rearing conditions (Hossain et al. 2024; Madhulika et al., 2025).

Among the promising candidates for functional feed additives, brown macroalgae have emerged as important natural sources of bioactive compounds with diverse health-promoting effects in cultured fish species (Naiel et al. 2021; Pourazad et al. 2024). Several studies have shown that dietary inclusion of brown algae can improve fish growth performance, enhance antioxidant enzyme activity, and stimulate immune responses such as, *Laminaria japonica* in *Micropterus salmoides* (Shen et al. 2025) and *Padina australis* in *Cyprinus carpio* (Sheikhzadeh et al. 2022). For instance, it had been previously reported that *Sargassum spp.* dietary administration in Nile tilapia significantly improved feed conversion ratio, lysozyme activity, and hepatic antioxidant status (Negm et al. 2021). In particular, *Dictyota dichotoma* is a brown seaweed rich in several bioactive compounds such as, phenols (24.1 ± 0.5 mg GAE g⁻¹), tannins (12.14 ± 0.2 mg GAE g⁻¹), and flavonoids (26.0 ± 5.8 mg RE g⁻¹), along with strong antioxidant activity as evidenced by DPPH (IC₅₀ = 458.24 ± 2.98 µg mL⁻¹) and H₂O₂ scavenging (IC₅₀ = 20.57 ± 13.82 µg mL⁻¹) (El-Sheekh et al. 2023). Also, FTIR-ATR analysis confirmed the presence of alkane, carboxylic acid, and aromatic amine functional groups, while GC–MS profiling identified n-hexadecenoic acid and tri-, tetra-, penta-, and octadecanoic acids as the major bioactive constituents (Velankanni et al. 2022). Together, the identified bioactive compounds and functional groups provide a mechanistic basis for the pronounced antioxidant and free radical scavenging activity of *Dictyota dichotoma*. Although the available data on the application of *Dictyota spp.* in aquafeeds remain limited, recent evidence suggests its potential in improving immune and antioxidant responses in various fish species such as *Danio rerio* (Mahmoudi et al. 2022) and *Clarias gariepinus* (Aderolu et al. 2025).

In addition to macroalgae, probiotics—especially spore-forming bacteria like *Bacillus* spp.—are widely utilized in aquaculture due to their ability to modulate gut microbiota, enhance nutrient utilization, and stimulate host immunity (Arungamol et al. 2025; El-dweny et al. 2025). Among them, *Bacillus coagulans* has gained attention for its stability under harsh environmental conditions and its capacity to confer functional benefits across different fish species (Fath El-Bab et al. 2022). In Nile tilapia, dietary inclusion of *B. coagulans* at 2 g/kg has been shown to improve serum lysozyme and complement C3 levels, elevate intestinal villus height, and remarkably improved the regulation of interleukin and growth related genes (Fath El-Bab et al. 2022). Similarly, dietary supplementation with *Bacillus coagulans* (1 × 10⁸ CFU/g) significantly enhanced immune gene expression, improved intestinal morphology, and beneficially modulated gut microbiota composition in crucian carp (*Carassius auratus*), leading to increased resistance against *Aeromonas veronii* infection (Hu et al. 2024).

Despite the proven individual benefits of brown algae (Abdelfatah et al. 2023) and probiotics (Hu et al. 2024; Omar et al. 2024), little is known about their potential synergistic effects when combined in aquafeeds. While blends of microalgae and probiotics have demonstrated enhanced gut morphology, immune activity, and growth in *Dicentrarchus labrax* (Yazici 2025) and *Litopenaeus vannamei* (Ajdari et al. 2025), studies specifically evaluating the combined impact of *B. coagulans* and brown macroalgae like *D. dichotoma* are lacking. Therefore, the current in vivo trial aimed to investigate the individual and combined effects of dietary *Bacillus coagulans* and *Dictyota dichotoma* on growth performance, feed utilization, immune responses, antioxidant status, body composition, and hepatic cytokine gene expression in common carp. Also, this study provides a novel contribution to functional feed development strategies that integrate algal and probiotic components for improved health and productivity in freshwater aquaculture.

## Materials & Methods

### Applied Additives

A probiotic commercial powder product containing *Bacillus coagulans* DSM 32,016 (Technospore^®^; Biochem Co., Germany) at 2.5 × 10⁹ CFU/g was incorporated as a safe dietary additive. Brown algae (*Dictyota dichotoma*) were hand-collected during low tide from the rocky intertidal zone of the Red Sea in Hurghada, Egypt (27°17′13″ N; 33°46′21″ E). Immediately after harvesting, the algae were rinsed twice with clean distilled water to remove epiphytes and debris, then placed in sterile plastic bags and transported to the laboratory. Upon arrival, specimens were washed thoroughly with freshwater, followed by multiple rinses with distilled water to eliminate residual impurities. Species identity was verified by a phycology specialist using standard morphological keys, and the handling process adhered to the methodology outlined by Deyab et al. (2016). The cleaned solid material was air-dried indoors at 25 ± 1.03 °C for five days, ground into a fine powder with an electric mill, and stored in airtight containers at 4 °C until use in diet formulation.

### Formulated Experimental Diets

The basal and experimental diet formulations were prepared following the protocols described by Jena et al. (1999), to meet and cover the full nutritional requirements of common carp (*Cyprinus carpio*) (Table 1). Diets were formulated using high-quality ingredients typically used in carp feeds to ensure balanced protein, lipid, vitamin, and mineral levels. Feed additives were incorporated by thoroughly mixing them with the ground feed ingredients to ensure uniform distribution. The mixture was then conditioned with water, pelleted using a mechanical pelletizer, and dried at ambient temperature before storage. After a full day of storage, the viable count of applied probiotic bacteria in the examined diets were confirmed employing the procedures outlined by Kumaree et al. (2015).

**Table 1:** Ingredient and formulated diet chemical analysis

Ingredients	CTR	BC	D_1_	D_2_	D_3_	BC*D_1_	BC*D_2_	BC*D_3_
Fish meal	280	280	280	280	280	280	280	280
Soybean meal	350	350	350	350	350	350	350	350
Wheat midds	120	118	117.5	115	110	115.5	113	108
Wheat flour	220	220	220	220	220	220	220	220
Fish oil	10	10	10	10	10	10	10	10
Vegetable oil	10	10	10	10	10	10	10	10
*Bacillus coagulanos*	0.0	2	0.0	0.0	0.0	2	2	2
*Dictyota dichotoma*	0.0	0.0	2.5	5	10	2.5	5	10
Premix	10	10	10	10	10	10	10	10
Total	1000	1000	1000	1000	1000	1000	1000	1000
Chemical Composition (%)								
Moi	10.89	10.89	10.89	10.89	10.89	10.89	10.89	10.89
DM	89.11	89.11	89.11	89.11	89.11	89.11	89.11	89.11
CP	39.89	39.98	39.88	39.89	39.89	39.98	39.88	39.89
CL	5.13	5.13	5.13	5.13	5.13	5.13	5.13	5.13
CF	2.55	2.56	2.51	2.52	2.55	2.56	2.51	2.52
Ash	7.78	7.96	8.08	8.09	7.78	7.96	8.08	8.09
GE (kJ/g)	1955.37	1952.85	1950.14	1950.03	1955.37	1952.85	1950.14	1950.03
NFE	44.65	44.37	44.40	44.37	44.65	44.37	44.40	44.37

^1^ Danish fish meal 60% protein obtained from Triple Nine Fish Protein, DK6700 Esbjerg, Denmark

^2^ Egyptian soybean flour 47.0% protein obtained from National Oil Co., Giza, Egypt

^3^ Vitamin premix (per kg of premix): thiamine, 2.5 g; riboflavin, 2.5 g; pyridoxine, 2.0 g; inositol, 100.0 g; biotin, 0.3 g; pantothenic acid, 100.0 g; folic acid, 0.75 g; para-aminobenzoic acid, 2.5 g; choline, 200.0 g; nicotinic acid, 10.0 g; cyanocobalamine, 0.005 g; a-tocopherol acetate, 20.1 g; menadione, 2.0 g; retinol palmitate, 100,000 IU; cholecalciferol, 500,000 IU

^4^ Mineral premix (g/kg of premix): CaHPO4.2H2O, 727.2; MgCO4.7H2O, 127.5; KCl 50.0; NaCl, 60.0; FeC6H5O7.3H2O, 25.0; ZnCO3, 5.5; MnCl2.4H2O, 2.5; Cu (OAc)2.H2O, 0.785; CoCl3.6H2O, 0.477; CaIO3.6H2O, 0.295; CrCl3.6H2O, 0.128; AlCl3.6H2O, 0.54; Na2SeO3, 0.03

^5^chemical analysis data were expressed as mean ± standard division (SD)

^6^ Nitrogen-Free Extract (calculated by difference) = 100 – (protein% + lipid% + ash% + fiber %)

^7^ Gross energy (GE) was calculated from NRC (2011) as 23.6 kJ/g, 39.4 kJ/g, and 17.2 kJ/g for protein, lipid, and carbohydrates, respectively

### Fish Welfare and Rearing Conditions

The feeding trial was conducted for eight consecutive weeks at a privately owned hatchery located in Kafr El-Sheikh Governorate, Egypt, under controlled field conditions. A total of 400 healthy common carp (*Cyprinus carpio*) fingerlings of uniform size and health status were acclimated for two weeks before the start of the experiment. Following acclimation, fish were individually weighed, randomly allocated into 40 floating hapas (each measuring 1 × 1 × 1 m and constructed from fine mesh netting), and stocked at a density of 10 fish per hapa. All hapas were suspended within the same earthen pond to ensure uniform environmental conditions and were continuously supplied with water from a single source (ground water).

The experimental design consisted of eight dietary treatments with five replicates each: (1) control diet without supplementation, (2) *Bacillus coagulans* at 2 g/kg, (3) *Dictyota dichotoma* at 2.5 g/kg (D_1_), (4) *D. dichotoma* at 5 g/kg (D_2_), (5) *D. dichotoma* at 10 g/kg (D_3_), (6) *B. coagulans* (2 g/kg) + *D. dichotoma* at 2.5 g/kg (BC × D_1_), (7) *B. coagulans* (2 g/kg) + *D. dichotoma* at 5 g/kg (BC × D_2_), and (8) *B. coagulans* (2 g/kg) + *D. dichotoma* at 10 g/kg (BC × D_3_). The inclusion levels of *B. coagulans* and *D. dichotoma* were selected according to effective dosages previously reported by Fath El-Bab et al. (2022) and Abdulrahman et al. (2019), respectively.

Water quality was closely monitored to maintain optimal rearing conditions for common carp. Throughout the trial, water temperature was maintained at 27 ± 1 °C, dissolved oxygen was kept above 5 mg/L via continuous water exchange and external paddle aeration, and pH was stabilized between 7.3 and 7.5. The natural photoperiod during the experimental period provided approximately 12 h light and 12 h dark daily. Fish were hand-fed twice daily (09:00 and 15:00 h) to apparent satiation, with feed amounts adjusted weekly according to biomass measurements to reduce waste and ensure consistent nutrient intake.

### Growth Traits

The growth metrics were tracked by weighing the surviving fish from each hapa in grams every two weeks until the end of the feeding trial. The initial body weight (IBW) was recorded as the average weight of the fish on day 0, and the final body weight (FBW) was recorded as the average weight on day 56. In details, body weight gain (BWG) was calculated as FBW minus IBW. Meanwhile, total consumed feed (TFI) per fish was determined by accurately measuring the amount of feed consumed. Specifically, uneaten feed was collected 30 min after feeding by siphoning, rinsed with freshwater to remove debris, oven-dried to a constant weight, and weighed. The dry weight of the recovered feed was subtracted from the total feed offered to estimate the actual feed intake. TFI was then divided by the number of fish to obtain the per-fish value. Feed conversion ratio (FCR) was calculated as TFI divided by BWG, while feed efficiency ratio (FER) was BWG divided by TFI. Daily weight gain percent (DWG%) was computed as (WG/days) ×100. Specific growth rate (SGR, %/day) was calculated as ([Ln (FBW) − Ln (IBW)]/days) ×100, and relative growth rate (RGR, %) as (BWG ÷ IBW) × 100.

### Specimens’ Collection Protocol

At the end of the feeding experiment, ten fish from each treatment (two fish per replicate) were randomly selected for sampling. Prior to sampling, all fish were fasted for 24 h to ensure stable physiological conditions. To eliminate handling-induced stress, the fish were gently netted and anesthetized with 2-phenoxyethanol (0.5 mL/L; Sigma, USA). Blood was collected from the caudal vein within 3 min of anaesthesia using sterile 1-mL syringes (Terumo, Mexico) and transferred into Eppendorf tubes without anticoagulant. The samples were centrifuged for 10 min to separate the serum, which was then stored at − 20 °C for subsequent biochemical, immunity, and antioxidant analyses. For gene expression studies, approximately 50 mg liver specimens were aseptically excised from each fish immediately after blood collection. The tissues were briefly rinsed in sterile phosphate-buffered saline (PBS) to remove excess blood, blotted dry on RNase-free filter paper, placed in sterile RNase-free microtubes, and snap-frozen in liquid nitrogen. The samples were then stored at − 80 °C until RNA extraction.

### Analysis Fish Flesh and Diet Composition

For proximate composition analysis, representative diet samples were collected after pelleting and drying, ground to a fine powder, and stored in airtight containers until analysis. Whereas, whole-body fish samples were obtained by randomly selecting three fish per replicate hapa at the end of the feeding trial. Meanwhile, chemical analyses of both the whole fish body and diet samples were performed to determine dry and organic matter, total moisture, crude protein, crude fat, and ash contents, following the standard methods of the AOAC (2005).

### Immune Parameters

Humoral immune responses were assessed by measuring serum immunoglobulin M (IgM) and immunoglobulin D (IgD) levels using fish-specific ELISA kits (Cusabio Biotech Co., Ltd., Wuhan, China; Catalog Nos.: MBS282651 for IgM and MBS2025555 for IgD), following the manufacturer’s instructions. Lysozyme activity was determined using a turbidimetric assay based on the lysis of *Micrococcus lysodeikticus*, as described by Ellis (1990). Complement component C3 activity was quantified using a fish-specific ELISA kit (MyBioSource; Catalog No.: 0–120-0009), in strict accordance with the provided protocol.

### Serum Antioxidant Features

To evaluate the redox status, serum concentrations of malondialdehyde (MDA), superoxide dismutase (SOD), catalase (CAT), and total antioxidant capacity (TAC) were determined using commercially available kits (MyBioSource and Abcam; Catalog numbers: MBS2700234 for MDA, MBS705758 for SOD, MBS726781 for CAT, and AB65329 for TAC). The assays followed the protocols of Uchiyama and Mihara (1978), Nishikimi et al. (1972), Goth (1991) and Amado et al. (2009), respectively. Furthermore, glutathione peroxidase (GPX) activity was measured with a specific kit (MyBioSource; Catalog No.: MBS2540412) according to the method of Faraji et al. (1987).

### Serum Biochemical Analysis

Serum albumin (ALB) and total protein (TP) concentrations were measured using colorimetric methods with a UV/VIS spectrophotometer (Opti Zen POP). Globulin (GLB) content was calculated by subtracting albumin from total protein values, and the albumin-to-globulin (A/G) ratio was obtained by dividing albumin by globulin. Hepatic function markers, including aspartate aminotransferase (AST) and alanine aminotransferase (ALT), were determined using diagnostic kits (BioMed Diagnostic, Egypt; Catalog No.: ABACM241035), following the protocols of Wilkinson et al. (1972) and Folin and Wu (1919). Renal function biomarkers, namely creatinine and uric acid, were quantified using colorimetric assay kits (BioMed Diagnostic, Egypt; Catalog Nos.: CRE105100 for creatinine and UA11909970 for uric acid), according to the manufacturer’s instructions.inson

### Gene Quantification Methods

Total RNA was extracted from approximately 50 mg of liver tissue using TRIzol reagent (iNtRON Biotechnology, Korea) following the manufacturer’s guidelines. To prevent RNA degradation or contamination, all procedures were carried out using RNase-free consumables, pre-cleaned benches, and gloves, with pipette tips and tubes certified as RNase-free. RNA purity and concentration were measured using a UV-Vis spectrophotometer (Quawell Q5000, USA), and only samples with an A260/A280 ratio between 1.8 and 2.0 were used for downstream applications. First-strand complementary DNA (cDNA) synthesis was performed with the SensiFAST™ cDNA Synthesis Kit (Bioline, UK). Quantitative real-time PCR (qRT-PCR) was conducted on a Stratagene MX3000P thermocycler using the SensiFAST SYBR Lo-Rox Kit (Bioline). Primers targeting immune-related genes (including, interleukin-6 (*IL-6*), tumor necrosis factor-2 alpha (*TNF-2α*), and interleukin-10 (*IL-10*) were designed using Primer2 software, meanwhile *β*-actin and GAPDH served as reference genes for normalization (Table 2). Also, primer sequences and their corresponding NCBI accession numbers are listed in Table 2 contents. The thermal cycling protocol consisted of an initial denaturation at 95 °C for 10 min, followed by 40 cycles of 95 °C for 15 s and 60 °C for 30 s, with a final extension at 85 °C for 5 min. Relative mRNA expression levels were determined using the 2^^−ΔΔCt^ method (Livak and Schmittgen 2001), ensuring that amplification efficiencies for all targets were approximately 100%.

**Table 2:** The primer sequencing of target selected genes applied for *q*-RT-PCR techniques

Gene	Forward 5′->3′	Reverse 5′->3′	Accession NO.
IL-10	ATGGGATACACATTTTTCTCTTCTGAGCAA	GCCTTCTGTTAAATTGAAATAAATTAC	NC_056607.1
IL-6	ATCAGCGGCGTAGCATTGAA	CGGTTGAGAGGAGGCTTTGT	NC_056615.1
TNF-2a	GAATACAAGGCCAGAAAGGATGACAC	GACCTTTTGAGTCGCTGCCTTC	NC_056601.1
GAPDH	CCTCCTTCCGCAGTAAGAGAA	TTTATGACCACAATTTGAGCCC	NM_001279552.1
β-Actin	TATGGCTAGAGCCAGGCAACCGGCTGAGTA	AATCCCAATAAAGTGCACATGTGTTCCGAA	XM_003443127

IL-10: interleukin-10 (anti-inflammatory cytokine); IL-6: interleukin-6 (pro-inflammatory cytokine); TNF-2a (TNF-α2): tumor necrosis factor alpha-2 (pro-inflammatory cytokine); GAPDH: glyceraldehyde-3-phosphate dehydrogenase (housekeeping gene); *β*-actin: beta-actin (housekeeping gene)

### Statical Technique

Statistical analyses were performed using SPSS software (version 24.0), and results are presented as mean ± pooled standard error (PSE). The collected and calculated data were analysis via two-way ANOVA to evaluate the main effects of dietary BC, dietary D, and their interaction. Data normality was verified using the Shapiro–Wilk test, and when significant effects were detected, Tukey’s post hoc test was applied for multiple comparisons. A probability value of *P* < 0.05 was considered statistically significant.

## Results

### Performance and Feed Efficiency

As presented in Table 3, dietary supplementation with *Bacillus coagulans* (BC), *Dictyota dichotoma* (D), and their interaction (BC×D) significantly influenced several growth and feed utilization parameters in common carp after 8 weeks. Supplementation with BC (2 g/kg) significantly improved feed intake (FI), feed conversion ratio (FCR), and feed efficiency ratio (FER) (*P* ≤ 0.001), but did not significantly affect final weight (FW), weight gain (WG), daily weight gain percentage (DWG%), relative growth rate (RGR), or specific growth rate (SGR) (*P* > 0.05). In contrast, *D. dichotoma* supplementation at different levels significantly influenced all measured performance parameters except initial weight, with the highest inclusion level (10 g/kg, D3) yielding the best results for FW (42.93 g), WG (40.00 g), FCR (1.01), FER (0.98), and SGR (5.96%/d) (*P* ≤ 0.001). Moreover, the interaction between BC and D significantly affected FI, FCR, DWG% and FER (*P* ≤ 0.05 or 0.001), where the combination of BC with 10 g/kg D (BC*D3) resulted in the lowest FI (38.59 g), lowest FCR (1.00), highest yielded DWG% (71%) and highest FER (1.03), indicating a synergistic effect. However, no significant interactions were observed for FW, WG, RGR, or SGR (*P* > 0.05). Overall, both BC and *D. dichotoma* improved feed utilization, while *D. dichotoma* exerted a stronger effect on growth performance, especially at higher inclusion levels, and their combination further enhanced feed efficiency outcomes.

**Table 3:** Performance, and efficiency of consumed feed in common carp fed diets supplemented with two different levels of *Bacillus coagulanos* (BC) (0.0–2 g kg^− 1^) and/or three different levels of *Dictyota dichotoma* (D) (2.5, 5 and 10 g kg^− 1^) for 8 weeks

Treatments	Parameters								
	IW(g)	FW(g)	WG(g)	DWG%	FI(g)	FCR(g/g)	RGR(g/g)	FER(g/g)	SGR(%/d)
BC effects									
CTR	3.00	41.0	37.10	66.25	52.80^a^	1.42^a^	92.51	0.70^b^	5.76
BC	2.97	39.37	36.40	65.00	41.00^b^	1.12^b^	92.46	0.88^a^	5.74
PSE	0.031	0.016	0.021	0.051	0.055	0.01	0.011	0.075	0.03
D effects									
D_1_	2.97	40.67^b^	37.70^c^	67.32^c^	40.64^b^	1.07^b^	92.70^ab^	0.92^b^	5.81^ab^
D_2_	2.93	41.93^ab^	38.96^a^	69.57^b^	50.62^a^	1.29^a^	92.92^ab^	0.76^c^	5.88^ab^
D_3_	2.97	42.93^a^	40.00^a^	71.43^a^	40.53^b^	1.01^b^	93.16^a^	0.98^a^	5.96^a^
PSE	0.06	0.051	0.053	0.10	0.28	0.013	0.02	0.008	0.06
Interaction BC*D effects									
BC*D_1_	2.97	39.30	36.33	64.88^c^	41.44^a^	1.14^a^	92.44	0.87^b^	5.74
BC*D_2_	2.93	41.33	38.40	68.57^b^	39.40^b^	1.02^b^	92.90	0.97^ab^	5.87
BC*D_3_	2.97	42.93	39.96	71.36^a^	38.59^b^	1.00^b^	93.08	1.03^a^	5.93
PSE	0.06	0.096	0.10	0.233	0.086	0.06	0.05	0.044	0.043
Two-way ANOVA (*P* value)									
BC	0.768	0.075	0.084	0.084	≤ 0.001	≤ 0.001	0.275	≤ 0.001	0.291
D	0.767	≤ 0.001	≤ 0.001	≤ 0.001	≤ 0.001	≤ 0.001	0.001	≤ 0.001	0.001
BC*D	0.708	0.585	0.617	0.017	≤ 0.001	≤ 0.001	0.815	≤ 0.001	0.835

CTR: control group; BC: group of fish fed a diet supplemented with 2 g/kg *Bacillus coagulans*; D1: group of fish fed a diet supplemented with 2.5 g/kg *Dictyota dichotoma* powder; D2: group of fish fed a diet supplemented with 5 g/kg *Dictyota dichotoma* powder; D3: group of fish fed a diet supplemented with 10 g/kg *Dictyota dichotoma* powder; BC*D1: group of fish fed a diet supplemented with 2 g/kg Bacillus coagulans and 2.5 g/kg Dictyota dichotoma powder; BCD2: group of fish fed a diet supplemented with 2 g/kg *Bacillus coagulans* and 5 g/kg *Dictyota dichotoma* powder; BC*D3: group of fish fed a diet supplemented with 2 g/kg *Bacillus coagulans* and 10 g/kg *Dictyota dichotoma* powder. IW, initial weight; FW, final weight; WG, weight gain; DWG%, daily weight gain percent; FI, feed intake; RGR, relative growth rate; FER, feed efficiency ratio; SGR, specific growth rate. Values within the same column having different superscripts are significantly different (*P* < 0.05). Data were presented as the mean ± pooled standard error

### Whole Fish Body Analysis

As shown in Table 4 contents, the whole-body proximate composition of common carp was significantly influenced by dietary inclusion of *Bacillus coagulans* (BC), *Dictyota dichotoma* (D), and their interaction. Supplementation with BC (2 g/kg) led to significant increases in ash, but reduced crude protein (CP) and ether extract (EE) levels compared to the control group (*P* ≤ 0.001), while indicating no significant influences on moisture (MOI), dry matter (DM), or organic matter (OM). In contrast, *D. dichotoma* supplementation significantly affected all estimated parameters (*P* ≤ 0.001), with the highest inclusion level (D_3_) resulting in elevated ash content (15.90%) and reduced OM (81.30%) and EE (14.93%). The 5 g/kg and 2.5 g/kg levels of D (D_2_ and D_1_) notably increased EE content to 17.15% and 16.72%, respectively. Furthermore, significant BC×D interactions (*P* ≤ 0.001 or 0.01) were observed for all parameters except OM. The combination of BC and 2.5 g/kg D (BC*D_*1*_*)* resulted the highest ash content (16.76%) and lowest CP (66.97%) and EE (14.01%), whereas BC*D2 reported in higher EE (16.00%) and intermediate CP (68.87%). These findings indicate that both BC and *D. dichotoma*, individually and in combination, modulate the body composition of common carp, with *D. dichotoma* exerting a more pronounced influence across all proximate parameters.

**Table 4:** Proximate body chemical composition in common carp fed diets supplemented with two different levels of *Bacillus coagulanos* (BC) (0.0–2 g kg^− 1^) and/or three different levels of *Dictyota dichotoma* (D) (2.5, 5 and 10 g kg^− 1^) for 8 weeks

Groups	Parameters %					
	MOI	DM	OM	Ash	CP	EE
BC effects						
CTR	77.74	22.14	82.08	11.96^b^	72.29^a^	14.32^a^
BC	77.82	22.17	81.93	16.82^a^	69.91^b^	12.25^b^
PSE	0.08	0.03	0.15	0.13	0.55	0.42
D effects						
D_1_	77.85^a^	22.21^b^	83.35^a^	12.95^b^	69.59^a^	16.72^a^
D_2_	76.70^ab^	23.39^a^	83.34^a^	11.98^bc^	69.83^a^	17.15^a^
D_3_	77.78^a^	22.37^ab^	81.30^b^	15.90^a^	68.81^ab^	14.93^b^
PSE	0.01	0.15	0.28	0.11	0.36	0.34
Interaction BC*D effects						
BC*D_1_	77.22^b^	22.73^a^	83.43	16.76^a^	66.97^c^	14.01^b^
BC*D_2_	77.43^ab^	22.59^a^	82.98	14.15^b^	68.87^b^	16.00^a^
BC*D_3_	77.51^a^	22.42^b^	81.13	14.61^b^	70.80^a^	13.94^b^
PSE	0.08	0.04	0.45	0.32	0.33	0.26
Two-way ANOVA (*P* value)						
BC	0.362	0.219	0.318	≤ 0.001	≤ 0.001	≤ 0.001
D	≤ 0.001	≤ 0.001	≤ 0.001	≤ 0.001	≤ 0.001	≤ 0.001
BC*D	≤ 0.001	≤ 0.001	0.749	≤ 0.001	≤ 0.001	0.002

CTR: control group; BC: group of fish fed a diet supplemented with 2 g/kg *Bacillus coagulans*; D1: group of fish fed a diet supplemented with 2.5 g/kg *Dictyota dichotoma* powder; D2: group of fish fed a diet supplemented with 5 g/kg *Dictyota dichotoma* powder; D3: group of fish fed a diet supplemented with 10 g/kg *Dictyota dichotoma* powder; BC*D1: group of fish fed a diet supplemented with 2 g/kg Bacillus coagulans and 2.5 g/kg Dictyota dichotoma powder; BCD2: group of fish fed a diet supplemented with 2 g/kg *Bacillus coagulans* and 5 g/kg *Dictyota dichotoma* powder; BC*D3: group of fish fed a diet supplemented with 2 g/kg *Bacillus coagulans* and 10 g/kg *Dictyota dichotoma* powder. MOI, moisture; DM, dry matter; OM, organic matter; CP, crude protein; EE, either extract. Values within the same column having different superscripts are significantly different (*P* < 0.05). Data were presented as the mean ± pooled standard error

### Immunological Aspects

As existing in Table 5, immune response parameters in common carp, including lysozyme (LYZ), complement component C3 (COMP-C3), immunoglobulin M (IgM), and immunoglobulin D (IgD), were significantly altered by dietary supplementation with *Bacillus coagulans* (BC), *Dictyota dichotoma* (D), and their interactions. Supplementation with BC (2 g/kg) significantly increased LYZ, COMP-C3, IgM and IgD levels (*P* ≤ 0.01 or 0.001) compared to the un-supplemented group. Similarly, *D. dichotoma* supplementation at all tested levels significantly enhanced all immune parameters (*P* ≤ 0.001), with the highest dose (10 g/kg, D_3_) yielding the greatest increases in LYZ (11.75 ng/ml), COMP-C3 (596.63 U/ml), IgM (31.05 ng/ml), and IgD (29.41 ng/ml). Significant interactions between BC and D (BC×D) were also observed for COMP-C3 (*P* ≤ 0.001), IgM (*P* = 0.003), and IgD (*P* ≤ 0.001), but not for LYZ. The combined treatment BC*D_3_ subjected the highest overall immune responses, particularly for LYZ (12.59 ng/ml), COMP-C3 (599.22 U/ml), IgM (34.73 ng/ml), and IgD (30.03 ng/ml), indicating a synergistic enhancement of immune function. These results confirm that both BC and *D. dichotoma*, individually and in combination, significantly enhance the innate and humoral immune responses in common carp, with the highest efficacy observed when both additives are used together at optimal inclusion levels.

**Table 5:** Immune responses in common carp fed diets supplemented with two different levels of *Bacillus coagulanos* (BC) (0.0–2 g kg^− 1^) and/or three different levels of *Dictyota dichotoma* (D) (2.5, 5 and 10 g kg^− 1^) for 8 weeks

Groups	Parameters			
	LYZ(ng/ml)	COMP-C3(U/ml)	IgM(ng/ml)	IgD(ng/ml)
BC effects				
CTR	7.09^b^	349.91^b^	25.63^b^	19.77^b^
BC	7.24^a^	352.56^a^	28.12^a^	20.11^a^
PSE	0.7	1.86	0.71	0.27
D effects				
D_1_	8.85^b^	412.5^c^	26.46^b^	19.92^b^
D_2_	8.52^b^	462.10^b^	27.38^b^	29.98^a^
D_3_	11.75^a^	596.63^a^	31.05^a^	29.41^a^
PSE	0.57	4.26	0.53	0.33
Interaction BC*D effects				
BC*D_1_	9.59^b^	464.70^b^	28.32^bc^	27.98^b^
BC*D_2_	10.04^b^	461.86^b^	29.69^b^	28.68^b^
BC*D_3_	12.59^a^	599.22^a^	34.73^a^	30.03^a^
PSE	0.46	11.76	0.63	0.66
Two-way ANOVA (*P* value)				
BC	0.006	≤ 0.001	0.005	≤ 0.001
D	≤ 0.001	≤ 0.001	≤ 0.001	≤ 0.001
BC*D	0.003	≤ 0.001	0.003	≤ 0.001

CTR: control group; BC: group of fish fed a diet supplemented with 2 g/kg *Bacillus coagulans*; D1: group of fish fed a diet supplemented with 2.5 g/kg *Dictyota dichotoma* powder; D2: group of fish fed a diet supplemented with 5 g/kg *Dictyota dichotoma* powder; D3: group of fish fed a diet supplemented with 10 g/kg *Dictyota dichotoma* powder; BC*D1: group of fish fed a diet supplemented with 2 g/kg Bacillus coagulans and 2.5 g/kg Dictyota dichotoma powder; BCD2: group of fish fed a diet supplemented with 2 g/kg *Bacillus coagulans* and 5 g/kg *Dictyota dichotoma* powder; BC*D3: group of fish fed a diet supplemented with 2 g/kg *Bacillus coagulans* and 10 g/kg *Dictyota dichotoma* powder. LYZ, lysozyme; COMP-C3, complement C3; IgM, immunoglobulin M; IgD, immunoglobulin D. Values within the same column having different superscripts are significantly different (*P* < 0.05). Data were presented as the mean ± pooled standard error

### Redox Status Features

As summarized in Table 6, dietary supplementation with *Bacillus coagulans* (BC), *Dictyota dichotoma* (D), and their interaction significantly modulated the antioxidant defensive mechanisms in common carp after 8 weeks. Specifically, BC supplementation (2 g/kg) led to a significant reduction in malondialdehyde (MDA) levels (*P* ≤ 0.001), as well as significant increases in glutathione peroxidase (GPX), total antioxidant capacity (TAC), catalase (CAT) and superoxide dismutase (SOD) activities (*P* ≤ 0.05 or 0.001). Herein, *D. dichotoma* supplementation significantly enhanced all antioxidant parameters (*P* ≤ 0.001), with the highest inclusion level (10 g/kg, D_3_). Specifically, the D_3_ group indicated lowest MDA (2.33 nmol/ml) and highest GPX (116.63 nmol/ml), TAC (124.92 ng/ml), CAT (12.31 U/ml), and SOD (14.95 U/ml) levels. Meanwhile, significant BC×D interaction effects (*P* ≤ 0.05 or 0.01) were observed for all estimated oxidative measurements. Exactly, the best overall antioxidant profile was recorded in the BC*D_3_ group, which exhibited the lowest MDA (2.17 nmol/ml) and highest levels of SOD (16.17 U/ml), GPX (118.50 nmol/ml), CAT (13.12 U/ml), and TAC (124.92 ng/ml).

**Table 6:** Antioxidant activities in common carp fed diets supplemented with two different levels of *Bacillus coagulanos* (BC) (0.0–2 g kg^− 1^) and/or three different levels of *Dictyota dichotoma* (D) (2.5, 5 and 10 g kg^− 1^) for 8 weeks

Groups	Parameters				
	MDA(nmol/ml)	GPX(nmol/ml)	TAC(ng/ml)	CAT(U/ml)	SOD(U/ml)
BC effects					
CTR	5.40^a^	85.48^b^	112.91^b^	9.97^b^	8.82^a^
BC	4.79^b^	90.97^a^	116.73^a^	10.04^a^	8.59^b^
PSE	0.45	3.1	2.4	0.28	0.4
D effects					
D_1_	4.42^ab^	100,50^c^	113.86^b^	9.85^b^	8.68^b^
D_2_	4.62^a^	102.65^b^	114.84^b^	10.90^b^	9.16^b^
D_3_	2.33^c^	116.63^a^	124.92^a^	12.31^a^	14.95^a^
PSE	0.33	1.13	0.73	0.69	0.29
Interaction BC*D effects					
BC*D_1_	3.43^a^	100.14^c^	115.07^c^	11.86^b^	9.73^b^
BC*D_2_	2.86^b^	102.20^b^	116.39^b^	12.31^b^	10.64^b^
BC*D_3_	2.17^c^	118.50^a^	124.92^a^	13.12^a^	16.17^a^
PSE	0.13	0.31	0.59	0.29	0.44
Two-way ANOVA (*P* value)					
BC	≤ 0.001	0.019	0.052	≤ 0.001	≤ 0.001
D	≤ 0.001	≤ 0.001	≤ 0.001	≤ 0.001	≤ 0.001
BC*D	0.003	0. 011	0.016	0.036	0.027

CTR: control group; BC: group of fish fed a diet supplemented with 2 g/kg *Bacillus coagulans*; D1: group of fish fed a diet supplemented with 2.5 g/kg *Dictyota dichotoma* powder; D2: group of fish fed a diet supplemented with 5 g/kg *Dictyota dichotoma* powder; D3: group of fish fed a diet supplemented with 10 g/kg *Dictyota dichotoma* powder; BC*D1: group of fish fed a diet supplemented with 2 g/kg Bacillus coagulans and 2.5 g/kg Dictyota dichotoma powder; BCD2: group of fish fed a diet supplemented with 2 g/kg *Bacillus coagulans* and 5 g/kg *Dictyota dichotoma* powder; BC*D3: group of fish fed a diet supplemented with 2 g/kg *Bacillus coagulans* and 10 g/kg *Dictyota dichotoma* powder. MDA, malonaldehyde; GPX, glutathione peroxidase; TAC, total antioxidant activity; CAT, catalase; SOD, super oxide dismutase. Values within the same column having different superscripts are significantly different (*P* < 0.05). Data were presented as the mean ± pooled standard error

### Blood Biochemical Parameters

Table 7 reveals that dietary supplementation with *Dictyota dichotoma* (D), *Bacillus coagulans* (BC), and their interaction significantly influenced various blood biochemical parameters in common carp after 8 weeks. In details, supplementation with D markedly increased total protein (TP), albumin (ALB), and globulin (GLOB) levels (*P* ≤ 0.001), with the highest values observed in fish fed 10 g/kg D (D_3_). Notably, D_3_-fed fish showed the highest TP (7.15 g/dl), ALB (4.05 g/dl), and GLOB (3.64 g/dl) alongside reduced liver enzyme activities (AST: 22.07 U/l, ALT: 37.80 U/l), suggesting improved liver function. Conversely, BC supplementation alone did not significantly affect protein fractions, but it significantly reduced AST (*P* ≤ 0.001) and ALT (*P* ≤ 0.01) levels, while increasing creatinine (CREA) and uric acid (UA) concentrations (*P* ≤ 0.01). In parallel, significant interaction effects (BC×D) were found for TP, GLOB, ALB/GLOB ratio, AST, CREA, and UA. Accurately, the combined treatment of BC with 10 g/kg D (BC*D_3_) yielded the most positive outcomes, showing the highest TP (7.81 g/dl), GLOB (3.64 g/dl), and ALB (4.16 g/dl), along with the lowest AST (20.89 U/l), ALT (36.10 U/l), CREA (0.33 mg/dl), and UA (0.02 mg/dl), indicating a synergistic improvement in systemic protein profile and renal-hepatic function.

**Table 7:** Blood biochemical parameters in common carp fed diets supplemented with two different levels of *Bacillus coagulanos* (BC) (0.0–2 g kg^− 1^) and/or three different levels of *Dictyota dichotoma* (D) (2.5, 5 and 10 g kg^− 1^) for 8 weeks

Treatments	Parameters							
	TP(g/dl)	ALB(g/dl)	GLOB(g/dl)	ALB/GLOB	AST(U/l)	ALT(U/l)	CREA(mg/dl)	UA(mg/dl)
BC effects								
CTR	5.10	2.38	2.72	0.90	25.89^a^	40.32^a^	0.38^b^	0.02^b^
BC	4.74	2.44	2.30	1.08	24.50^b^	39.65^b^	0.46^a^	0.08^a^
PSE	0.19	0.27	0.46	0.28	0.08	0.34	0.03	0.01
D effects								
D_1_	5.25^c^	3.04^b^	2.20^b^	1.40^b^	25.55^a^	39.84^a^	0.37^a^	0.05^a^
D_2_	6.10^b^	3.74^ab^	2.36^b^	1.58^a^	24.73^a^	40.57^a^	0.40^a^	0.04^b^
D_3_	7.15^a^	4.05^a^	3.64^a^	1.31^b^	22.07^b^	37.80^b^	0.31^b^	0.02^b^
PSE	0.39	0.22	0.16	0.18	0.33	0.87	0.02	0.01
Interaction BC*D effects								
BC*D_1_	5.18^c^	3.48	1.70^bc^	2.06^a^	24.66^a^	38.89	0.46^a^	0.07^a^
BC*D_2_	6.04^b^	3.74	2.30^b^	1.63^ab^	22.28^b^	39.11	0.40^b^	0.05^a^
BC*D_3_	7.81^a^	4.16	3.64^a^	1.14^b^	20.89^c^	36.10	0.33^b^	0.02^b^
PSE	0.14	0.09	0.12	0.17	0.19	0.76	0.02	0.02
Two-way ANOVA (*P* value)								
BC	0.511	0.134	0.385	0.056	≤ 0.001	0.005	≤ 0.001	0.005
D	≤ 0.001	≤ 0.001	≤ 0.001	≤ 0.001	≤ 0.001	≤ 0.001	≤ 0.001	0.001
BC*D	0.011	0.427	0.012	0.023	≤ 0.001	0.763	0.006	0.047

CTR: control group; BC: group of fish fed a diet supplemented with 2 g/kg *Bacillus coagulans*; D1: group of fish fed a diet supplemented with 2.5 g/kg *Dictyota dichotoma* powder; D2: group of fish fed a diet supplemented with 5 g/kg *Dictyota dichotoma* powder; D3: group of fish fed a diet supplemented with 10 g/kg *Dictyota dichotoma* powder; BC*D1: group of fish fed a diet supplemented with 2 g/kg Bacillus coagulans and 2.5 g/kg Dictyota dichotoma powder; BCD2: group of fish fed a diet supplemented with 2 g/kg *Bacillus coagulans* and 5 g/kg *Dictyota dichotoma* powder; BC*D3: group of fish fed a diet supplemented with 2 g/kg *Bacillus coagulans* and 10 g/kg *Dictyota dichotoma* powder. TP, total protein; ALB, albumin; GLOB, globulin; ALB/GLOB ratio, albumin- globulin ration; AST, aspartate aminotransferase; ALT, alanine aminotransferase; CREA, creatinine; UA, uric acid. Values within the same column having different superscripts are significantly different (*P* < 0.05). Data were presented as the mean ± pooled standard error

### Gene Regulation

**Fig. 1:**
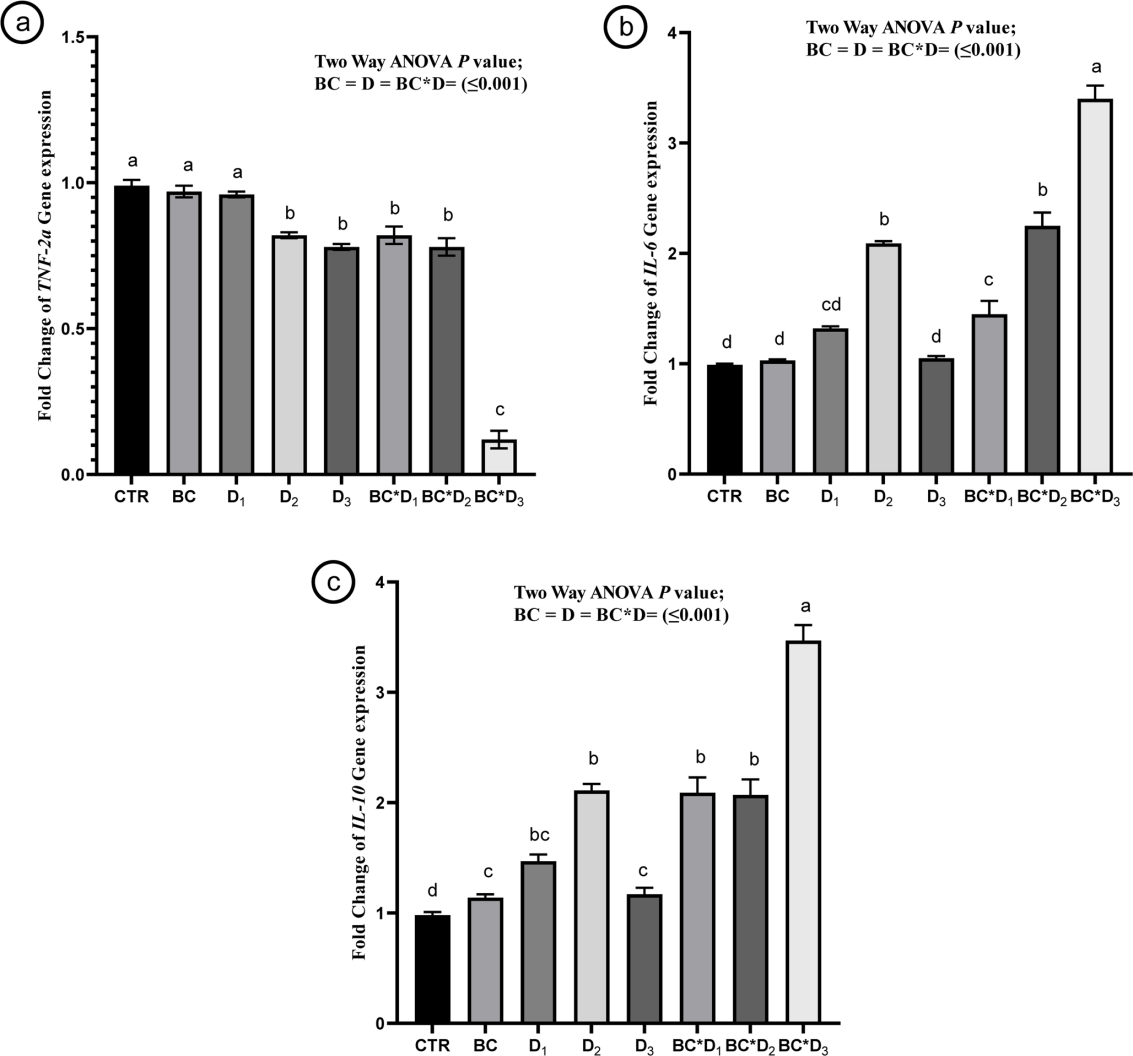
Expression levels of Tumor Necrosis Factor-alpha, *TNF-α* (**a**), Interleukin-6, *IL-6* (**b**), and Interleukin-10, *IL-10* (**c**) in common carp fed diets supplemented with two levels of *Bacillus coagulans* (CTR and BC; 0.0–2 g kg⁻¹) and/or three levels of *Dictyota dichotoma* (D; 2.5, 5, and 10 g kg⁻¹) for 8 weeks. Data are presented as mean ± mean standard error. Statistical analysis was performed using two-way ANOVA, and different superscripts above columns indicate significant differences (*P* < 0.05)

Relative mRNA expression levels of inflammatory and cytokine-related genes (*TNF-2α*, *IL-6*, and *IL-10*) in the liver of common carp after 8 weeks of feeding diets supplemented with *Bacillus coagulans* (0.0–2 g/kg) and/or *Dictyota dichotoma* powder (2.5, 5, or 10 g/kg) are presented in the content of Fig. 1. The obtained results indicated that dietary inclusion of *B. coagulans* alone (BC) resulted in a slight downregulation of *TNF-2α* and a notable increase in *IL-10* expression (*P ≤* 0.001). Furthermore, supplementation with *D. dichotoma* (D_1_–D_3_) led to dose-dependent upregulation of *IL-6* and *IL-10*, with the highest expression in the D_2_ group (*P ≤* 0.001). In addition, co-supplementation (BC*D_1_, BC*D_2_, BC*D_3_*)* exhibited synergistic effects, particularly in the BC*D_3_ group, where *TNF-2α*, *IL-6*, and *IL-10* were significantly upregulated (*P ≤* 0.001).

## Discussion

The increasing interest towards aquaculture nutrition has highlighted the benefits of employing multiple feed additives that operate through different physiological pathways (El-dweny et al. 2025). Combining functional feed additives like probiotics and brown algae can provide synergistic advantages. Probiotics such as *Bacillus coagulans* primarily improve gut health, microbial balance, and nutrient digestibility (Fath El-Bab et al. 2022), while brown algae like *Dictyota dichotoma* are rich in bioactive polysaccharides, minerals, and antioxidants that can enhance metabolism, boost immune function, and improve feed palatability (Aderolu et al. 2025; Mahmoudi et al. 2022). Investigating the combined effects of these additives is essential for optimizing fish growth performance and feed efficiency, particularly in economically valuable species like common carp (*Cyprinus carpio*). These strategies could help reduce dependence on synthetic growth promoters and antibiotics, thus promoting sustainable aquaculture practices.

The current findings indicate that dietary supplementation with *Bacillus coagulans* (BC), *Dictyota dichotoma* (D), and their combination exerted notable effects on the growth and feed utilization performance of common carp (*Cyprinus carpio*). Specifically, supplementation with BC at 2 g/kg significantly enhanced feed intake (FI), feed conversion ratio (FCR), and feed efficiency ratio (FER). The current findings aligning with previous reports demonstrated that *Bacillus* probiotics improve nutrient digestion and absorption by modulating gut microbiota and producing extracellular enzymes (Wu et al. 2012; Zhang et al. 2021). Conversely, BC alone did not significantly alters final weight (FW), weight gain (WG), or growth rate indices, which may suggest that the growth-promoting effects of BC in carp are more closely linked to improved nutrient utilization rather than direct stimulation of growth, as similarly observed in rainbow trout (Raeeszadeh et al. 2025).

Alternatively, dietary inclusion of *D. dichotoma* significantly improved almost all measured growth and feed efficiency measurements, with the highest inclusion level (10 g/kg) producing the best outcomes in FW, WG, FCR, FER, and SGR. These results are consistent with earlier studies showing that brown seaweeds, rich in bioactive polysaccharides (e.g., fucoidan, alginate) and phenolic compounds, enhance feed palatability, stimulate digestive enzyme secretion, and promote nutrient assimilation in *Dicentrarchus labrax* fish (Fonseca et al. 2023) and white spotted snapper, *Lutjanus stellatus Akazaki* juveniles (Zhu et al. 2017). The observed synergistic effects in the BC × D treatments, particularly BC*D_3_, on feed efficiency metrics further suggest that combining a probiotic with a bioactive-rich brown algae creates complementary modes of action of probiotics enhancing gut microbial balance and algae-derived compounds boosting digestive and metabolic processes (Ringø et al. 2016). Exactly, this synergy is likely enabled by the prebiotic features of polysaccharides derived from brown algae, such as alginates and fucoidans (Nazarudin et al. 2020). These molecules enhance the viability and colonization of entire probiotics in the gut (Khanzadeh et al. 2025). Also, balanced microbial environment may stimulate the secretion of digestive enzymes, improve intestinal structure, and increase nutrient digestibility, leading to better feed utilization and growth rates (El-dweny et al. 2025). Furthermore, bioactive compounds found in brown algae, including phenolics and terpenoids, might eliminate intestinal redox stress and inflammation, creating a more favorable gut environment for probiotic activity (Vidhya et al. 2019). While BC primarily improved feed utilization, *D. dichotoma* had a more significant effect on growth performance. Combined, they resulted in the most efficient feed conversion.

In the same direction, evaluating the whole-body proximate composition of fish is essential, as it reveals whether absorbed nutrients are stored as proteins, lipids, or minerals (Eissa et al. 2024). The current whole-body structure results indicate that both BC and *D. dichotoma* individually significantly increased ash content while decreasing protein and lipid levels in a dose-dependent manner. In contrast, the combined BC*D_3_ group remarkably improved protein retention levels while diminishing ash and lipid content. The current findings align well with previous studies reporting that probiotic supplementation can increase fish flesh ash content while modulating protein and lipid content via improved mineral absorption and promoted metabolic regulation (Atef et al. 2024; Hassaan et al. 2021). Similarly, including brown macroalgae in the diet has been shown to increase ash content due to its high mineral availability, while also reducing lipid deposition by regulating lipid metabolism through bioactive compounds (Cadar et al. 2025; Pandey et al. 2025). The interaction effects of *Bacillus coagulans* and high levels of *D. dichotoma* demonstrated their influence on mineral absorption and enhanced lipid deposition, as previously reported for multi-additive feeding strategies (Ringø et al. 2022). However, alterations in the nutrient composition of flesh are essential, as reduced lipid content and balanced protein deposition can enhance the nutritional value of aquaculture products (Shahin et al. 2023). This not only promotes healthier options for consumers but also supports sustainable production goals (Naiel et al. 2023).

On the other side, measuring lysozyme and complement C3 provides insights into innate humoral defenses, while IgM and IgD indicate adaptive immune responses, which include both systemic and mucosal protection (Thepot et al. 2021). Together, these markers allow for the evaluation of dietary and environmental impacts on overall immune competence and disease resistance in fish (Naiel et al. 2023). In this study, the immune response parameters in *Cyprinus carpio* were significantly modulated by *B. coagulans*, *D. dichotoma*, and their combinations. The highest levels of lysozyme activity, complement C3, IgM, and IgD were found in the BC*D_3_ group following by D_3_ group, indicating a strong synergistic effect. These findings are consistent with previous reports showing that *Bacillus spp.* enhance serum lysozyme, complement activity, and immunoglobulin levels in carp and tilapia (He et al. 2023; Yang et al. 2019), as well as evidence that brown algae supplementation improves humoral and mucosal immunity in fish (Shen et al. 2025). Notably, the probiotic strain *Bacillus coagulans* has been shown to indirectly support the immune system by enhancing gut microbial balance and promoting the competitive exclusion of pathogens through its bacterial cell wall components and metabolites (Ringø et al. 2022). In contrast, *Dictyota dichotoma* serves directly as both an immunostimulant and an antioxidant due to its high levels of polysaccharides (such as fucoidan and laminarin), polyphenols, and fatty acids, which stimulate both innate and adaptive immunity (Saeed et al. 2021; Vijayabaskar et al. 2012). Additionally, gene expression data further confirmed the anti-inflammatory properties of *B. coagulans* by increasing *IL-10* levels while slightly downregulating *TNF-2α*. In contrast, *D. dichotoma* supplementation increased *IL-6* and *IL-10* in a dose-dependent manner. Meanwhile, co-supplementation (BC*D_3_) resulted in significant upregulation of both *IL-6*, and *IL-10* and downregulation of *TNF-2α* genes. Similar results related to the upregulation of cytokines through the influence of probiotics in rainbow trout (Gorgoglione et al. 2013; Xu et al. 2024) or algal bioactive molecules in *Sparus aurata* (Reyes-Becerril et al. 2013). Previous studies have shown that *B. coagulans* promotes gut barrier integrity and reduces inflammation by promoting beneficial microbiome, such as *Akkermansia*, and increasing the production of short-chain fatty acids (SCFAs) (Zheng et al. 2022). Additionally, it modulates signaling pathways like *NF-κB*, resulting in a widespread reduction of intestinal inflammatory responses (Song et al. 2024). Meanwhile, the observed synergistic effects of combining *B. coagulans* with *Dictyota dichotoma* are likely due to the algae’s rich phytochemical composition, particularly terpenes, which have strong antioxidant and anti-inflammatory properties through inhibiting the production of inflammatory mediator pathways, including nitric oxide (NO) (Agatonovic-Kustrin et al. 2018; Martić et al. 2023).

In fish, the antioxidant defensive pathways is closely linked to immune competence, as it limits oxidative damage, preserves cellular homeostasis, and sustains the activity of immune cells during stress or infection (Naiel et al. 2022). In this trial, dietary supplementation with *Bacillus coagulans* (BC), *Dictyota dichotoma* (D), and their combination significantly modulated antioxidant responses in common carp. In details, BC supplementation reduced lipid peroxidation and enhanced catalase (CAT) and superoxide dismutase (SOD) activities, supporting previous findings that probiotic *Bacillus* species improve antioxidant status by modulating gut microbiota, generating antioxidant metabolites, and reducing free radical production (Shahraki et al. 2025; Taherpour et al. 2023). Conversely, BC showed no significant effect on glutathione peroxidase (GPX) or total antioxidant capacity (TAC), suggesting its main contribution lies in enhancing enzymatic radical detoxification rather than overall antioxidant capacity.

In contrast, *D. dichotoma* supplementation significantly improved all antioxidant parameters, with the highest inclusion producing the greatest benefits. These effects are attributed to its rich bioactive profile, particularly phloro-tannins, fucoidans, alginates, carotenoids, and polyphenols, which scavenge free radicals and upregulate antioxidant enzyme expression via the *Nrf*_*2*_*–Keap1* pathway (Matin et al. 2024). Such mechanisms not only enhance oxidative stress resistance but also strengthen immune function, as previously reported in fish fed brown algae extracts (Mota et al. 2023). A significant BC × D interaction for lipid peroxidation and SOD activity revealed synergistic benefits when BC was combined with high-dose *D. dichotoma*, likely due to the complementary actions of probiotic-mediated nutrient assimilation and immune modulation alongside the direct antioxidant activity of algal bioactive molecules. Similar synergistic responses between probiotics and macroalgae have been documented in aquaculture, resulting in reduced oxidative stress, improved immunity, and greater resilience to environmental stressors (Ajdari et al. 2025).

In addition, blood biochemical profiles are widely recognized as reliable indicators of fish health, reflecting nutritional condition, immune status, and organ functionality (Abdelghany et al. 2020). In the present feeding experiment, dietary inclusion of *Dictyota dichotoma* notably enhanced total protein, albumin, and globulin concentrations, accompanied by a decrease in liver enzyme activities (AST and ALT). These improvements are likely linked to the diverse bioactive compounds present in brown algae, such as phlorotannins, fucoidans, alginates, carotenoids, and essential amino acids (Deyab et al. 2016; El-Sheekh et al. 2023). Phlorotannins and fucoidans exert strong antioxidant and anti-inflammatory effects, protecting liver cells from oxidative damage and maintaining membrane stability, which could account for the observed reduction in AST and ALT levels (Barbosa et al. 2017). Furthermore, fucoidans and alginates may facilitate protein metabolism by enhancing nutrient absorption and stimulating hepatic protein synthesis (Li et al. 2023a, b). Comparable improvements in serum protein profiles and liver enzyme activity have been reported in Nile tilapia (Negm et al. 2021) and common carp (Galindo et al. 2022) supplemented with brown algae, highlighting their hepatoprotective and immune-supportive roles (Dong et al. 2019).

Besides, *Bacillus coagulans* supplementation alone primarily reduced AST and ALT levels, indicating hepatoprotective effects likely mediated via modulation of gut microbiota and enhancement of short-chain fatty acid production, which can influence liver metabolism and systemic antioxidant defensive mechanisms (Hoseinifar et al. 2024). Although BC did not significantly alter protein fractions, it increased creatinine and uric acid levels, suggesting a role in modulating nitrogen metabolism, potentially through microbial fermentation and improved amino acid availability (Li et al. 2023a, b). Importantly, significant BC × D interactions were observed for protein fractions, liver enzymes, and renal markers, with combined supplementation yielding the most favourable outcomes. The synergistic effect likely arises from complementary mechanisms. In details, BC enhances gut microbial balance and nutrient absorption, while *D. dichotoma* bioactive derivates directly reduce oxidative stress, modulate inflammatory pathways, and stimulate hepatic protein synthesis. Such probiotic–algae combinations have previously been shown to improve serum biochemical indices, support liver and kidney function, and enhance overall health in cultured fish (Ahmadifar et al. 2023; Soltani et al. 2019).

## Conclusion

This study demonstrated that the dietary co-supplementation of *Bacillus coagulans* (2 g/kg) and *Dictyota dichotoma* (10 g/kg) synergistically improved performance, feed utilization, and protein retention in common carp (*Cyprinus carpio*). Specifically, the combined treatment notably enhanced growth and feed efficiency indices, liver and kidney health, and elevated both innate and humoral immune responses. Additionally, it strengthened antioxidant defense pathways and upregulated key immune-related genes, including *IL-6*, *IL-10*, and *TNF-2α*. Besides, *D. dichotoma* alone at a high-level improved growth and feed efficiency, which *B. coagulans* primarily enhanced feed utilization with limited effect on growth. This underscores the potential of integrating probiotics with macroalgae as functional feed additives in aquaculture. Therefore, future research should focus on investigating the long-term effects under commercial farming conditions, measuring their influence on fish flesh quality criteria, and exploring the modulation of gut microbiota in relation to metabolomic profiling. Such efforts will help validate and support the sustainable application of this synergistic dietary strategy to enhance fish health and productivity.

## Data Availability

Available under reasonable request from the corresponding author.
